# Contribution of community health workers to the treatment of common illnesses among under 5-year-olds in rural Uganda

**DOI:** 10.1186/s12936-022-04316-1

**Published:** 2022-10-21

**Authors:** Fred Bagenda, Andrew Christopher Wesuta, Geren Stone, Moses Ntaro, Palka Patel, Jessica Kenney, Stephen Baguma, David Santson Ayebare, Shem Bwambale, Michael Matte, Peter Chris Kawungezi, Edgar Mugema Mulogo

**Affiliations:** 1 Department of Community Health, Mbarara University of Science and Technology, P.O Box 1410, Mbarara, Uganda; 2 Center for Global Health, Massachusetts General Hospital, 125 Nashua Street, Boston, MA 02114 USA; 3 Bugoye Health Centre, PO Box 149, Kasese, Uganda

**Keywords:** Common illnesses, Community Health Worker, Uganda, Under-five children

## Abstract

**Background:**

The control of malaria, pneumonia, and diarrhoea is important for the reduction in morbidity and mortality among children under 5 years. Uganda has adopted the Integrated Community Case Management strategy using Community Health Workers (CHWs) to address this challenge. The extent and trend of these three conditions managed by the CHWs are not well documented. This study was done to describe the epidemiology and trends of the three common illnesses treated by the CHWs in Bugoye Sub-County in rural Uganda.

**Methods:**

A retrospective review of monthly morbidity data for children less than 5 years of age for the period April 2014–December 2018 for CHWs in rural Bugoye Sub-County in Kasese district, Uganda was done. The total number reviewed was 18,430 records. The data were analysed using STATA version 14.

**Results:**

In total male were 50.2% of the sample, pneumonia was the highest cause of illness among the infants (< 1 year), while malaria was the highest among the children 1 year–59 months. Infection with a single illness was the commonest recorded cause of presentation but there were some children recorded with multiple illnesses. All the CHWs were managing the three common illnesses among children under 5 years. The trend of the three common illnesses was changing from malaria to pneumonia being the commonest. Children aged 12–24 months and 25–59 months were at 2.1 times (95% CI 1.7–2.4) and 5.2 times (95% CI 4.6–5.9), respectively, more likely to get malaria but less likely to get pneumonia and diarrhoea.

**Conclusion:**

Community Health Workers in rural Uganda are contributing significantly to the management of all the three commonest illnesses among under-5 years-old children. The trend of the commonest illness is changing from malaria to pneumonia. Children under 1 year are at a higher risk of getting pneumonia and diarrhoea and at a lower risk of getting malaria.

## Background

Malaria, pneumonia, and diarrhoea are still among the commonest causes of childhood morbidity and mortality globally in, sub-Saharan Africa and in Uganda [1, 2]. Volunteer Community Health Workers (CHWs) are community members who are not paid and are selected by fellow community members to provide accurate health information, counsel, mobilize communities and provide linkage for curative, prevention, health promotion, and care services. The World Health Organization (WHO) estimates that 57 countries in Asia and Africa including Uganda are using the Integrated Community Case Management (ICCM) strategy for the management of childhood illnesses in the community [2–7].

The Ministry of Health (MoH) in Uganda established Community Health Workers in 2001 and the integrated Community Case Management guidelines in 2010 to improve access to Universal Primary Health Care. Their role and contribution to the community management of the three commonest childhood illnesses have been proven to be quite crucial and successful especially in the rural areas that have limited access to health care [2, 7–12]. The WHO and UNICEF in 2006 developed a manual on the management of the sick child by CHWs with various case studies and models including the Homapak^®^ (malaria treatment) strategy in Uganda and these were demonstrated to have positive outcomes for the specific illnesses [7, 13, 14].

Using the Uganda MoH training guidelines, Community Health Workers (CHW/VHTs) are trained for 5 days during the initial training and later trained for additional 5 days on the Integrated Community Case Management (ICCM) full package by the Uganda Ministry of Health National Training Staff. Community Health Workers in Uganda under the Integrated Community Case Management (ICCM) strategy are involved in testing for malaria using Rapid Diagnostic Test (RDT) kits, assessing and treating of pneumonia and diarrhoea, and other prevention and health promotion activities in the community under the supervision of trained health workers at the health facility in the catchment area [3, 7, 15–19].

Community Health Workers report quarterly on all these indicators to the National District Health Information Software 2 (DHIS2). There has been limited data for long-term trends of these major cases seen by the CHWs over years in Uganda. It is important to know this because it helps the health providers to prioritize service delivery based on information. There is limited documentation of the trends of the common illnesses managed by Community Health Workers in the typical rural community within Bugoye Sub-County. This study focused on documenting the above gaps in Kasese district, Southwestern Uganda.

## Methods

The aim was to determine the epidemiology and a trend of the three most common illnesses among children less than 5 years of age for the period April 2014–December 2018 in rural Bugoye Sub-County in Kasese district, Uganda. A retrospective review of monthly morbidity reporting data of the CHWs for children less than 5 years of age was done. The data contained information on age, sex, diagnosis (malaria, pneumonia, and diarrhoea), and their respective treatment. Cleaning, validating, and analysis of this Integrated Community Case Management data was done for the period April 2014–December 2018. This is data collected every month at Bugoye Health Centre III by the CHWs in the catchment area supervised by the health facility.

### Study setting

Bugoye Health Centre III is situated in Bugoye Sub-County, which is located in Kasese district in southwestern Uganda. The population served by the Health Centre is approximately 46,124 of which 9225 are children under 5 years of age, it has 7650 households in 35 villages. This data included five villages in 2014 and 2015, eight villages in 2016, 2017, and 2018 when the ICCM programme was gradually being rolled out.

### Data collection

Data from the monthly Integrated Community Case Management reporting forms were aggregated and entered into a database in Epi-Data software from April 2014–December 2018. The data were then cleaned with comparison to paper records in case of a mismatch between reporting month and visit date or potential inaccuracy. This data set is available for any required review.

### Data analysis

The data set was exported to STATA 14 and analyses were done for the demographic characteristics, epidemiology, and trends of the cases that are seen and managed by the CHWs and the proportions of the prevalence of the three most common illnesses among the under-5 years-olds. Bivariate logistic regression for odds of the association was also done with the demographic characteristics (sex and age categories) and the common illnesses for an association between the demographic characteristics and the common illnesses. The independent variables included age category and sex, and the outcome was malaria cases, pneumonia cases, and diarrhoea cases.

### Ethical issues

This study was approved by the Research Ethics Committee of Mbarara University of Science and Technology and permission was got from the Kasese District administration to analyze and use the facility data. The analysis of the data was anonymous so consent for the individual cases was not done as they had identification numbers that were randomly allocated and kept anonymous. (Study number: 06/03–17).

## Results

### Demographic characteristics of children managed by the Community Health Workers

The total number of child encounters of the CHWs reviewed was 18,430 and males were 50.2% (9192) females were 49.8% (9105). These children were from eight villages and four parishes from Bugoye Sub-County in the Kasese district, details in Table 1.

**Table 1:** Sex distribution for the three illness treated by CHWs

	Male n (%)	Female n (%)	Total
2014	864 (49.6)	879 (50.4)	1743
2015	1441 (48.8)	1514 (51.2)	2955
2016	2016 (50.9)	1948 (49.1)	3964
2017	2772 (51.2)	2637 (48.8)	5409
2018	2099 (49.7)	2127 (50.3)	4226
	9192 (50.2)	9105 (49.8)	18297

Amongst the three diagnosed illnesses managed by the CHWs, pneumonia was the highest cause of illness among the infants (< 1 year) while malaria was the highest cause of illness among the children 1 year to 59 months, details in Table 2.

**Table 2:** Illness episodes among the different age categories

	Age category		
Diarrhoea	0–11 months	12–59 months	Total
Pneumonia	1338 (32.0)	3768 (21.1)	5106
Malaria	2080 (49.7)	6196 (34.6)	8276
Total	765 (18.3)	7926 (44.3)	8691
	4183	17890	

**Fig. 1:**
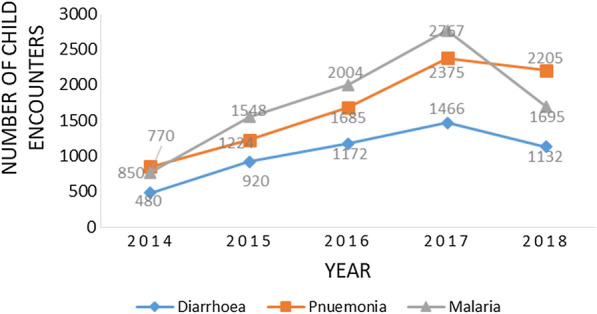
Five-year trend of the three common illnesses managed by CHWs

The three common illnesses of malaria, pneumonia, and diarrhoea were prevalent every month and throughout the years from 2014 to 2018. Single infection with one of the three illnesses was a common occurrence and malaria was the commonest illness while diarrhea was the least common throughout the review, details in Fig. 1.

**Fig. 2:**
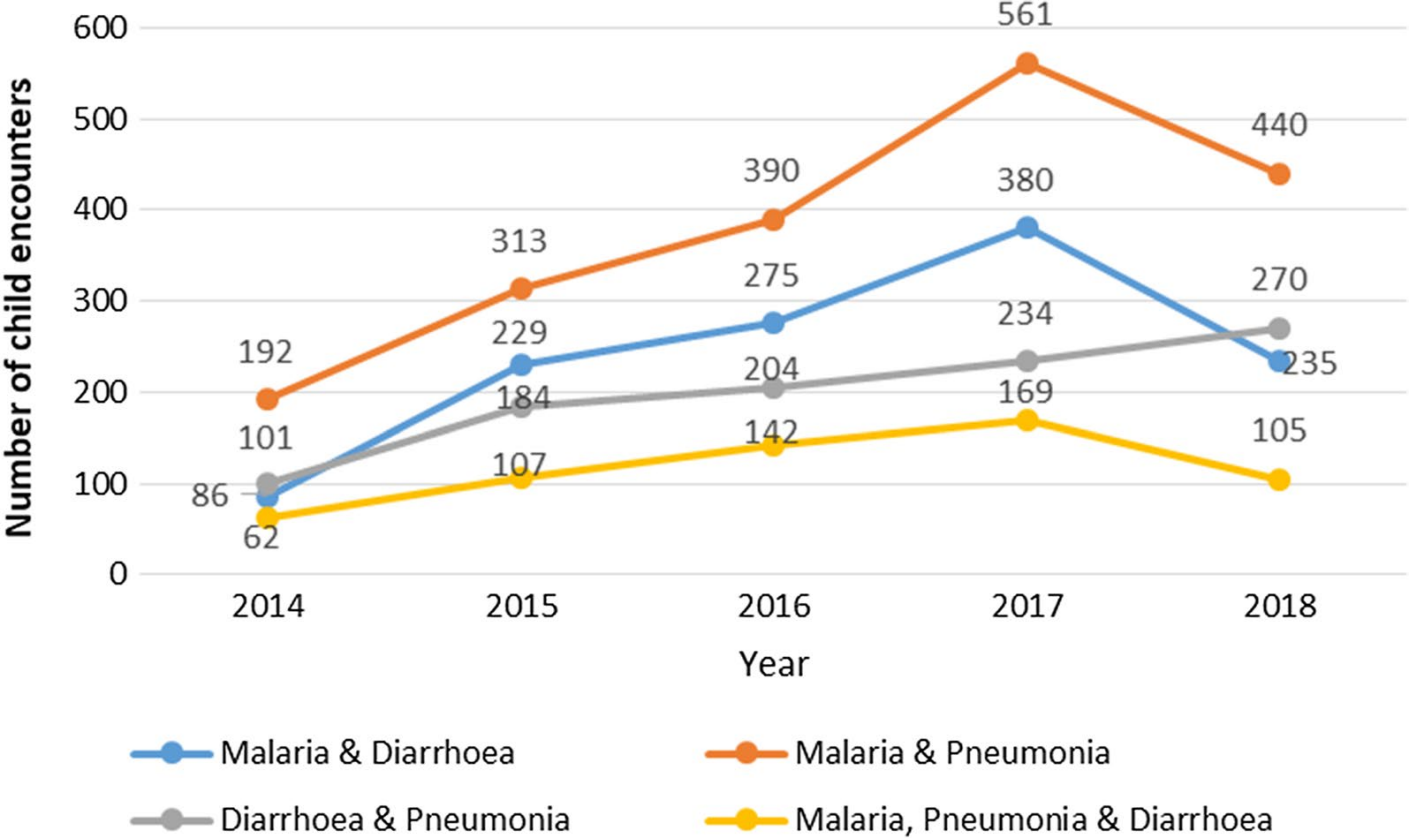
Multiple illness trends of the three conditions managed by CHWs

Multiple illnesses were prevalent every month of the year and over the whole study period. Combined illness with malaria and pneumonia was the commonest while the combination of malaria, pneumonia, and diarrhoea was the least common. Figure 2 shows the trends of multiple illnesses.

Being less than 1 year of age was associated with a higher risk of getting diarrhoea OR 0.8 CI 0.72–0.85 compared to “12–24” months of age and also higher risk of pneumonia OR 0.7 CI 0.60–0.71 compared to “12–24” months of age but lower risk of getting malaria, see Table 3.

**Table 3:** Associations of being under 1 year of age and having diarrhoea, pneumonia, or malaria

Disease condition	OR	p-value	95% CI
Diarrhoea (Under 1-year reference)			
12–24 months	0.8	< 0.001	0.72–0.85
25–59 months	0.4	< 0.001	0.37–0.44
Pneumonia (Under 1-year reference)			
12–24 months	0.7	< 0.001	0.60–0.71
25–59 months	0.4	< 0.001	0.36–0.42
Malaria (Under 1 reference)			
12–24 months	2.1	< 0.001	1.86–2.36
25–59 months	5.2	< 0.001	4.64–5.87

## Discussion

Malaria, pneumonia, and diarrhoea remain the three commonest causes of illness in Bugoye Sub-County for the period of 2014 to 2018 just like the other parts of Africa and Uganda which had similar documentation [1, 17, 20]. This current study showed that although most children had single infections, twenty-five percent had 2 or all 3 illnesses for the three commonest illnesses of malaria pneumonia, and diarrhoea. The findings are similar to six other sub-Saharan countries in Africa including Uganda [20, 21].

The treatment of children under 5 years of age for malaria, pneumonia, and diarrhoea are performed by each of the active and trained CHWs in each of the eight villages in Bugoye Sub-County for the ones that seek for treatment from them. This finding is similar to other countries, including Uganda, that adopted the Integrated Community Case Management (ICCM) in Africa and some regions in Southeast Asia [3–5, 8, 9, 16, 17, 19, 22, 29]. Community Health Workers are an important resource in the management of these illnesses [28].

For the period of the study that is April 2014–December 2018, out of the 24,000 and more children assessed and treated there was no reported death. These potentially good outcomes reflect similar findings of other studies of CHWS treatment for these three common illnesses [2, 4, 12, 14, 23–25]. This could also mean that the CHWs can identify very sick children with danger signs and refer them promptly.

The 5-year trend for the three commonest illnesses among children under 5 years of age in this study had malaria highest followed by pneumonia then diarrhoea during the first 4 years. This changed with illness due to malaria dropping drastically to about the same level as diarrhoea. This may be attributed to the malaria control interventions that have been strengthened over the last 10 years in Uganda and this has been evidenced by the gradual reduction in malaria test positivity and a drop in all-cause under-five malaria mortality (137–64/1000) over the last 10 years in Uganda in the 2018 WHO World Malaria Report and fact sheet of USAID [26, 27]. The 5-years trend of the multiple infections has malaria and pneumonia as the highest followed by malaria and diarrhoea, then diarrhoea and pneumonia and then the triple infection least this is consistent with a study in a Nigerian hospital in 2018 [12].

### Limitations

Because of the limited capacity of the CHWs on the understanding of the importance of reporting the data was not complete but this was addressed by cross-checking with the health facility data during the process of cleaning and validating the data. The data at the health facility is a combination of the data from the CHWs and that of the patients seen at the facility only data of the CHWs was analysed for this study.

## Conclusion

Community Health Workers are involved in the management of all the three commonest illnesses in Bugoye Sub-County, which are malaria, pneumonia, and diarrhoea. These illnesses sometimes present as multiple infections with various combinations of pneumonia, malaria, and diarrhoea. The trends of the three commonest illnesses among children under 5 years of age are changing with pneumonia now becoming the commonest cause of illness since 2017.

Children less than 1 year of age were associated with a lower risk of having malaria but a higher risk of having pneumonia or diarrhoea compared to children 1–5 years of age. Given the changing disease profile especially in children under 1 year, the focus of health interventions should put more pronounced emphasis on addressing the prevention and control of pneumonia among these vulnerable age groups of under 1-year-old. There needs to be monitoring of trends over time to ensure the Community Health Worker programme remains current with prevailing health conditions, and there is also importance of continuous monitoring and evaluation as part of the programme.

## Data Availability

All data supporting the study findings are contained in the paper. There are no restrictions to the data sources, however full details to the data may be accessed on reasonable request from the corresponding author.

## Abbreviations

CHW: Community Health Worker
VHT: Village Health Team)
CI: Confidence interval
DHIS: District Health Information System
ICCM: Integrated Community Case Management
OR: Odds Ratio
P-value: Probability
RDT: Rapid Diagnostic Test
UNICEF: United Nations Children’s Fund
USAID: United States Agency for International Development
WHO: World Health Organization
